# Assessing value-for-money in maternal and newborn health

**DOI:** 10.1136/bmjgh-2017-000310

**Published:** 2017-07-28

**Authors:** Aduragbemi Banke-Thomas, Barbara Madaj, Shubha Kumar, Charles Ameh, Nynke van den Broek

**Affiliations:** 1 Centre for Maternal and Newborn Health, Liverpool School of Tropical Medicine, Liverpool, Merseyside, UK; 2 Keck School of Medicine, University of Southern California, Los Angeles, California, USA

**Keywords:** Health Economics, Health Services Research, Health Systems Evaluation, Obstetrics

## Abstract

Responding to increasing demands to demonstrate value-for-money (VfM) for maternal and newborn health interventions, and in the absence of VfM analysis in peer-reviewed literature, this paper reviews VfM components and methods, critiques their applicability, strengths and weakness and proposes how VfM assessments can be improved. VfM comprises four components: economy, efficiency, effectiveness and cost-effectiveness. Both ‘economy’ and ‘efficiency’ can be assessed with detailed cost analysis utilising costs obtained from programme accounting data or generic cost databases. Before-and-after studies, case–control studies or randomised controlled trials can be used to assess ‘effectiveness’. To assess ‘cost-effectiveness’, cost-effectiveness analysis (CEA), cost-utility analysis (CUA), cost-benefit analysis (CBA) or social return on investment (SROI) analysis are applicable. Generally, costs can be obtained from programme accounting data or existing generic cost databases. As such ‘economy’ and ‘efficiency’ are relatively easy to assess. However, ‘effectiveness’ and ‘cost-effectiveness’ which require establishment of the counterfactual are more difficult to ascertain. Either a combination of CEA or CUA with tools for assessing other VfM components, or the independent use of CBA or SROI are alternative approaches proposed to strengthen VfM assessments. Cross-cutting themes such as equity, sustainability, scalability and cultural acceptability should also be assessed, as they provide critical contextual information for interpreting VfM assessments. To select an assessment approach, consideration should be given to the purpose, data availability, stakeholders requiring the findings and perspectives of programme beneficiaries. Implementers and researchers should work together to improve the quality of assessments. Standardisation around definitions, methodology and effectiveness measures to be assessed would help.

**Key questions**

**What is already known about this topic?**

- In maternal and newborn health, there have been increasing calls by stakeholders and donors for value-for-money (VfM) assessments of what are often quite complex interventions.
- VfM has been variably defined. However, the consensus is that VfM is an approach which aims at better use of resources for the wider good of people.
- The components of VfM include economy, effectiveness, efficiency and cost-effectiveness.

**What are the new findings?**

- Alternative approaches to strengthen VfM assessments are proposed: combine either a cost-effectiveness analysis or a cost-utility analysis with a detailed cost analysis and an effectiveness study or use either a cost-benefit analysis or social return on investment analysis independently.
- To provide critical contextual information to better understand and interpret VfM assessments, it is important to also assess and incorporate cross-cutting themes such as equity, sustainability, scalability and cultural acceptability of the intervention.

**How might this influence practice?**

- Researchers and implementers need to work closely together to ensure VfM is robustly and comprehensively assessed.
- Systematic sharing of experiences and best practices between organisations is important to ensure that the best quality assessments are conducted. Agreement and standardisation around definitions, methodology and effectiveness measures to be assessed would help to improve quality of VfM assessments.

## Introduction

**Figure 1:**
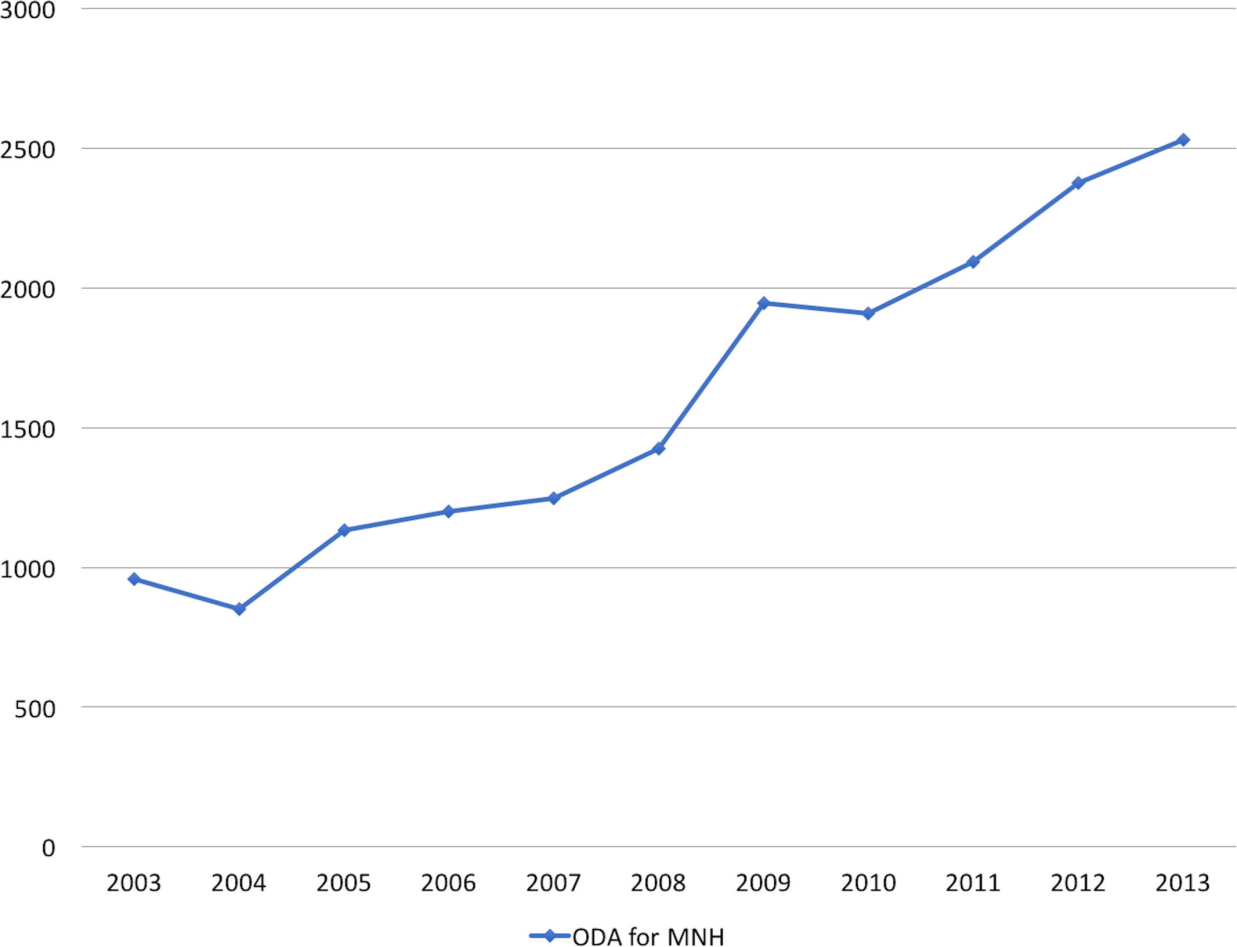
Overseas development assistance (ODA) for maternal and newborn health, 2003–2013.

There has been increasing demand by governments and international agencies for implementers and researchers to demonstrate value-for-money (VfM) of global health interventions.^1 2^ Specifically, in maternal and newborn health (MNH), where the focus is on improving pregnancy experience and outcomes for mothers and their newborns throughout the continuum of care, calls for stronger accountability and performance monitoring to ensure VfM have been made by multiple stakeholders.^3^ In low-income and middle-income countries (LMICs) where the burden of mortality and morbidity is highest, this call comes against the backdrop of increasing donor disbursements (US$2500 million based on a 2013 estimate) for MNH interventions (figure 1).^4^ There is, however, still limited information regarding the best approach in assessing VfM.

This paper reviews the different VfM definitions that have been put forward and the components and methods used to assess each of them. The applicability, strengths and limitations of each method were critiqued in light of MNH interventions, and considerations required in choosing an approach for VfM assessments are highlighted.

### What is value-for-money?

VfM generally refers to a product or service being worth the money spent on it. This concept of making good use of money is not novel and has been embedded in assessments of several health interventions implemented in the public sector,^1 2^ where it is often used as a synonym for ‘cost-effectiveness’.^5^ However, within the international development field, VfM generally refers to a broader concept encompassing economy, efficiency and effectiveness, in addition to cost-effectiveness.^5^

The Organisation for Economic Co-operation and Development describes VfM as ‘the optimum combination of whole-life cost and quality to meet the user's requirement’.^5^ New Zealand Aid describes VfM as ‘achieving the best possible development outcomes over the life of an activity relative to the total cost of managing and resourcing that activity and ensuring that resources are used effectively, economically, and without waste’.^6^ The National Audit Office in the UK describes VfM as ‘the optimal use of resources to achieve the intended outcomes’.^7^ Finally, the UK Department for International Development describes VfM as ‘the best use of resources to achieve intended sustainable outcomes and impact’.^8^ These descriptions show that VfM consists of several interlinked components. Altogether, the consensus is that VfM is not a tool or a method, but rather an approach to provide evidence for better resource allocation.^5^

**Figure 2:**
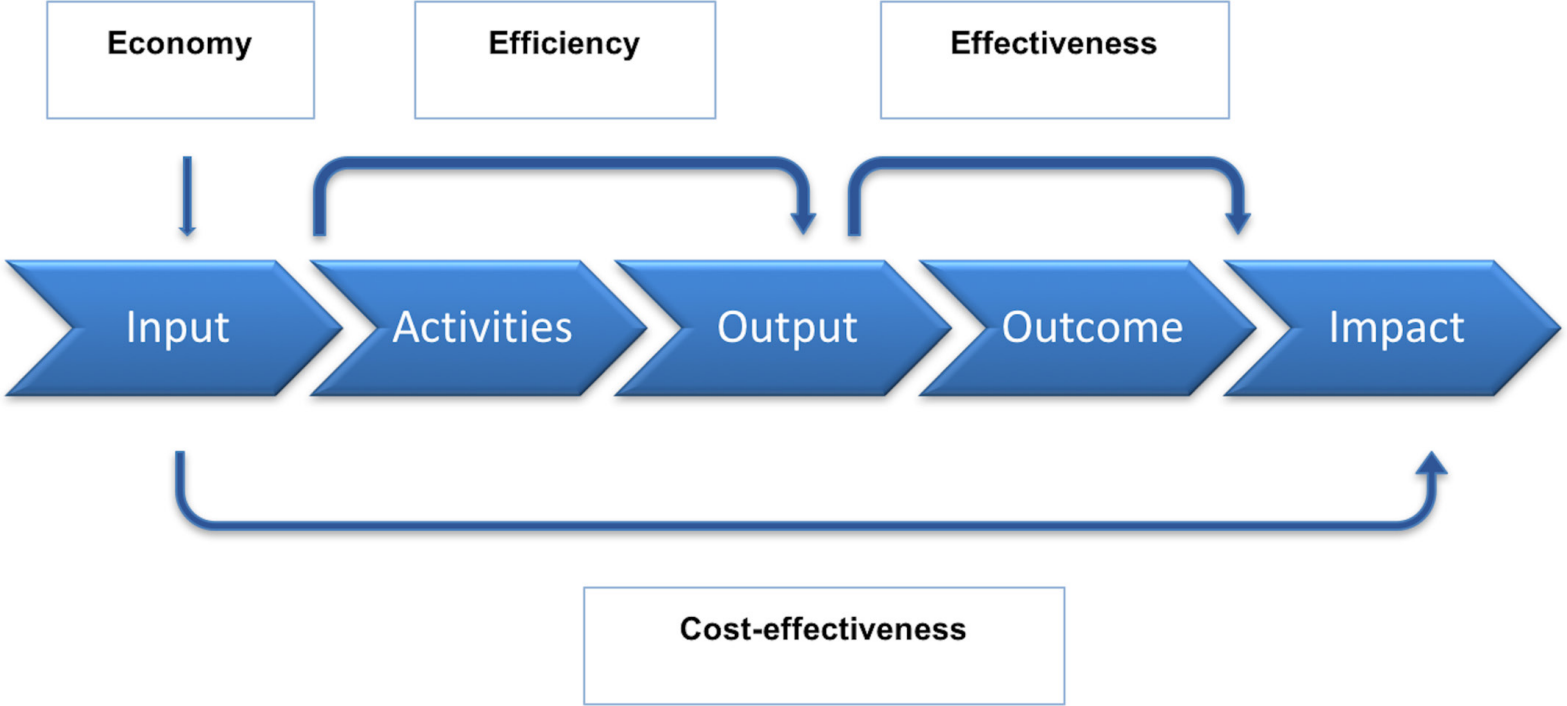
‘Economy, effectiveness and efficiency (3Es) and cost-effectiveness (CE)’ framework for value-for-money.

To better describe VfM, the economy, effectiveness and efficiency (3Es) and cost-effectiveness (CE) framework was proposed. This framework is linked to a logical chain of events which reflects the intervention's theory of change (figure 2).^8^ The VfM components address four key questions: Are implementers or funders buying the inputs of appropriate quality and at the right price? (economy), How well are implementers converting the inputs into outputs? (efficiency), How well are the outputs generated from an intervention achieving the desired outcomes? (effectiveness) and How much impact does an intervention achieve relative to the inputs that have been invested? (cost-effectiveness).^8^

### How can VfM be assessed?

Several methods can be used to assess the components of the VfM framework. An aggregation of these methods provides insight into how VfM can be assessed. As no examples of a VfM analysis are found in peer-reviewed literature, a hypothetical case study (*Mother and Baby Programme A*) has been provided to illustrate the VfM assessment process (box 1).

#### Economy and efficiency

Economy is not just about the cheapest option but also about purchasing high-quality inputs at the best price.^8^ Economy is assessed using a detailed cost analysis^9^ and based on financial rather than economic costs. Financial costs are described as ‘explicit costs’ covering actual implementation costs which can be direct or indirect. A direct cost is a price that can be completely attributed to the production of a cost object (such as a project). Indirect costs such as administration and overhead costs are not directly accountable to a cost object. Both direct and indirect costs may be either fixed or variable depending on whether they respond directly and proportionally to changes in volume purchased or sold. For example, in the case study, cost of training manuals is a direct cost (box 1). The unit cost for each manual may remain the same despite the number or manuals printed (fixed) or may change, for example, unit cost decreases as the quantity printed increases (variable). Economic costs, on the other hand, are viewed as more comprehensive, since in addition to the actual costs paid (financial costs) these also include ‘implicit costs,’ such as opportunity costs (the ‘cost of the alternative forgone’, eg, the cost of the time taken off work to attend a training).^10^ Furthermore, economic costs have to be annualised over the project lifetime at a discount rate, since the future value of money spent today is generally presumed to be greater than its present value.^10^ Both financial and economic costs have been used in MNH cost analyses.^11 12^ In the case study, financial costs were used (box 1). Although financial costs are more relevant for VfM assessments, there is value in estimating economic costs, as these could form the basis for economic evaluations in which cost-effectiveness of interventions are assessed.^13^

To generate the cost data needed for VfM assessments, assessors have the choice of either the bottom-up (ingredient) or the top-down (expenditure) approach.^14^ Unlike the top-down approach which breaks down total ‘expenditure’ into component costs (C_Total_=>C_1_+C_2_+C_3_), the bottom-up approach builds-up the ‘ingredients’ to estimate the total cost (C_1_ +C_2_+C_3_ =>C_Total_), as utilised in the case study (box 1). Both health economists and MNH experts who have conducted costing exercises recommend the bottom-up approach, which utilises micro-costing methods in identifying and valuing each resource required for a specific intervention.^10 13 15^ This approach allows for analysts and policymakers to check and verify each individual component included in an analysis and make decisions on where to save money without compromising on quality.^13 15^

Cost data can be gathered from programme accounting data as in the case study (box 1) and by Saronga *et al*^13^ or it can be collected from existing generic cost databases.^15^ A commonly used cost database is the WHO-CHOICE database developed by the WHO as the underlying framework for the CHOosing Interventions that are Cost-Effective (CHOICE) project.^16^ It is considered good practice to state the currency and year in which the cost data were collected. These costs can be subsequently converted to International Dollars (I$) using the purchasing power parity conversion factors, as was done by McPake *et al*.^17^ Such a conversion allows for ease of comparison across similar interventions and models.^18^

Efficiency which can be viewed as productivity and builds on economy refers to the cost of inputs per output produced. Efficiency is generally estimated using simple arithmetic, dividing the total costs (economy) by the output generated. This requires assessing whether the costs of inputs per outputs is comparable with similar interventions, as was done by Trémolet *et al*.^19^ For example, some MNH studies reporting cost analyses of emergency obstetric care training conducted in LMICs, also estimated the costs/training/participant,^11 20^ which is an indicator of efficiency.^19^ (box 1) When the cost currency and year in which the study was conducted are stated, conversion to I$ can be done and comparisons can then be made to establish which intervention/strategy is more efficient.^18^

Both economy and efficiency are within the direct control of the programme implementers and funders since they make the decision on how the funds are allocated and spent. As such they can make the choice(s) on how to achieve economy and efficiency. These choice(s) and the rationale that guided them are required to fully demonstrate these VfM components. Efforts should be focused on optimising economy and efficiency either by decreasing the inputs that produce a specified output or by increasing the outputs derived from the input.^21^

Additional consideration regarding the quality of the input(s) should be detailed in a supportive narrative for ‘economy’.^19^ Such rigour should be part of the standard procurement process, which would allow assessors to evaluate if they have purchased inputs at best possible price, when quality, quantity and availability considerations are made. For efficiency, meeting the project targets may be taken as a proxy-indicator, as such, project delays and reasons for not meeting the objectives can be highlighted.^19^ In the case study, existing government structures were used to achieve project targets (box 1).

Box 1 **Maternal and newborn health value-for-money (VfM) hypothetical case study—‘Mother and Baby Programme A’**

**Project title:** Improving availability of emergency obstetric care (EmOC).

**Background:** A grant was received from an international donor to reduce maternal mortality by increasing availability of EmOC in a designated district, which was known to have the least number of skilled providers per population and the highest maternal mortality ratio in the country. This was a 1-year long project. As part of the project mandate, the lead institution was required to embed VfM indicators in their management systems and conduct a VfM assessment of the intervention at the end of the project life cycle. Specifically, the intervention involved training midwives on context-specific best practices for EmOC. Background data on number of skilled providers and pregnancy outcomes (newborn and maternal mortality) were collected at the start of the project (baseline).

**Economy:** Using an ingredient approach which reviewed all the various components purchased towards the project implementation; the total programme expenditure was US$100 000. The cost data were based on project accounting records. When data were not clear, more detail was gathered during interviews with finance managers. Cost of training included the component costs of training manuals, venue, equipment and travel for national and international facilitators. The most cost-intensive part of the expenditure was cost relating to travel of the international facilitators. However, the facilitators were working on a voluntary basis. No per diems were paid to trainees. Each 5-day training costs US$5000.

**Efficiency:** The target to train 400 midwives by the end of the project was met. These midwives were trained at a cost of $250 per trained midwife. Working together with the Ministry of Health helped to ensure that the appropriate staff were trained and efforts were not duplicated.

**Effectiveness:** Compared to baseline (year before the intervention), there were 50 fewer maternal deaths in the year following the trainings. When disaggregated, most significant reduction in maternal deaths occurred in subdistrict A where most of the maternal deaths in the district previously occurred. Similarly, there were 400 fewer stillbirths. The maternal case fatality rate was 0.6% compared to 1.5% at baseline.

**Cost-effectiveness:** Cost-effectiveness analysis was used. A decision tree was used to compare costs and benefits at baseline and follow up. It cost US$2000 and US$250 to save each additional maternal life and prevent stillbirths, respectively.

**Overall VfM assessment:** The training was conducted in the area with the greatest need and when compared to other similar capacity building interventions (referenced elsewhere), it yielded higher efficiency and effectiveness and was more cost-effective. The training was deemed culturally relevant and useful by the key stakeholders as it was designed to be context-specific for low-resource settings. Overall, the project was considered to have offered VfM although it was considered important to explore more sustainable implementation models beyond the lifetime of the project.

### Effectiveness and cost-effectiveness

Effectiveness and cost-effectiveness are intricately linked. Both are dependent of external factors which may not be controllable by the implementers.^8^ Effectiveness demonstrates capacity of the intervention to deliver the intended outcome,^21^ while cost-effectiveness describes the amount of input required to deliver this outcome.^8^

Cost-effectiveness analysis (CEA), cost-utility analysis (CUA) and cost-benefit analysis (CBA) have been conventionally used to demonstrate ‘cost-effectiveness’. However, more recently and across different areas of global health, there has been growing interest in a new method, social return on investment (SROI).^1^ Most MNH cost-effectiveness studies have used CEA or CUA, while CBA and SROI are less frequently used^22–26^ (box 2). All four analytical tools provide some form of ‘cost-effectiveness’ ratio as an output of analysis. Across these tools, the definition of ‘cost’ is essentially the same; however, the manner of accounting for effectiveness differs^9^ (table 1).

**Table 1:** Comparison of methods for assessing cost-effectiveness

Cost-effectiveness analysis (CEA)	Cost-utility analysis subtype of CEA	Cost-benefit analysis (CBA)	Social return on investment (SROI)
Main objective			
Compare costs and outcomes of alternatives within the same domain	Compare costs and outcome of alternatives within the same domain	Assess worthwhileness of the investment made in implementing an intervention	Assess worthwhileness of the investment made in implementing an intervention, from the perspective of stakeholders
Accounting for costs			
Monetary value. Multiple sources can be explored, including direct and indirect costs as well as different perspectives	Monetary value Multiple sources can be explored, including direct and indirect costs as well as different points of view	Monetary value Multiple sources can be explored, including direct and indirect costs as well as different perspectives	Monetary value Multiple sources can be explored, including direct and indirect costs as well as different perspectives
Accounting for benefits			
Benefits referred to as ‘effectiveness’, which is reported as ‘natural units’ such as life years gained.	Benefits referred to as ‘utility’, which is reported as quality adjusted life years gained/disability adjusted life years averted/healthy life years gained	Benefits include both health and non-health outcomes, which are reported as monetary value or welfare benefit. Benefits that are difficult to monetise are listed	Benefits include both health and non-health outcomes, including social, economic and environmental outcomes. Both positive and negative effects are accounted for. Benefits are reported as monetary value or welfare benefit, using financial proxies to show monetary value of benefits that cannot be easily monetised.
Level of application			
Single intervention level	Single intervention level	Single or multiple intervention(s), project or programme level	Single or multiple intervention(s), project, programme, policy or organisation level
Time line of analysis			
Retrospective or prospective	Retrospective or prospective	Retrospective or prospective	Retrospective (evaluative type) or prospective (forecast type)
Discounting of future value			
Applicable	Applicable	Applicable	Applicable
Stakeholder engagement			
Not required	Not required	Not required	Multiple stakeholders required
Theory of change			
Not required for conduct	Not required for conduct	Not required for conduct	Required for conduct
Main output of analysis			
Incremental cost-effectiveness ratio (ICER). Sensitivity analysis is applicable	ICER. Sensitivity analysis is applicable	Benefit-cost ratio (BCR), economic internal rate of return, net present value (NPV), break-even point. Sensitivity analysis is applicable	SROI ratio, NPV, payback period. Sensitivity analysis is applicable
Interpretation of main output of analysis			
Intervention with higher cost-effectiveness ratio is better	Intervention with higher cost-effectiveness ratio is better	BCR >1 is worthwhile investment	SROI ratio >1 is worthwhile investment
Relevance			
Priority setting and resource allocation	Priority setting and resource allocation	Priority setting and resource allocation	Priority setting, resource allocation, stakeholder relationship building, accountability framework and management tool

Box 2 **Summary of published examples of maternal and newborn health cost-effectiveness studies**

**Cost-effectiveness analysis** (Somigliana *et al*^25^)

**Background:** Study assessed cost-effectiveness of a 24-hour ambulance service in Oyam district of northern Uganda.

**Costs data:** Collected from the perspective of the district health provider. Direct costs included costs of the ambulance, fuel and drugs were included and indirect costs like personnel costs.

**Benefits data:** Over a 3-month period, data were collected in a prospective fashion. Two experienced obstetricians managed all referred cases and made their assessments independently. Benefits were estimated based on number of years saved for mother and newborn (natural units), using the local life expectancy table as benchmark. Benefits of non-obstetrical referrals and those relating to quality of life and disability were excluded.

**Analysis:** Benefits to costs ratio estimated. Using WHO thresholds, the authors assessed cost-effectiveness of the intervention. Three per cent discount was applied. Sensitivity analysis was conducted.

**Results:** Ninety-two obstetric referrals were recorded. Eleven (12%) were considered effective, corresponding to 611.7 years saved. Cost per year saved was US$15.82. The intervention remained extremely cost-effective with the sensitivity analysis, as the cost per year saved value remained lower than WHO US$30 benchmark for defining very attractive interventions.

**Cost-utility analysis** (Broughton *et al*^26^)

**Background:** Study assessed cost-effectiveness of an intervention that used a quality improvement strategy known as improvement collaborative to increase compliance of health workers with known standardised high impact interventions including emergency obstetric and newborn care in Niger.

**Costs data:** Based on project accounting records. For costs not retrievable from this source, information was gathered during interviews with clinic managers and members of the quality improvement teams.

**Benefits data:** Collected from participating facilities using a before and after design. Over a 5-month period (for baseline) and 3-month period (for follow up), 26 months apart. Benefits were facility-based maternal care outcomes. Postpartum haemorrhage and acute management of third stage labour indicators were recorded by reviewing partographs of spontaneous vaginal birth in participating sites each month. Emergency neonatal care was based on average compliance with immediate newborn care standards. Disability-adjusted life years (DALY) outcome measures were calculated using a 3% discount rate as well as disability weights for moderate and severe anaemia, 20.046 and 0.093, respectively. Age weighting was also used. Study did not take into account maternal health changes that may have occurred independently of the intervention.

**Analysis:** Calculations were based on the assumptions that average age of mother at delivery was 26 and average life expectancy was 54. A decision tree was used to compare costs and benefits at baseline and follow-up. Incremental cost-effectiveness ratio (ICER) was calculated.

**Results:** Average delivery cost decreased from US$35 at baseline to US$28 at follow-up. The collaborative ICER was calculated as US$147/DALY averted.

**Cost-benefit analysis** (Lappalainen *et al* 22)

**Background:** Study reported a CBA that compared ‘no-screening’ and ‘screening’ alternatives for primary toxoplasmosis during pregnancy in Finland.

**Costs data:** Collected from available Finnish sources that detailed cost of screening.

**Benefits data:** Collected from existing sources. Monetised benefit data included direct (medical and institutional care) and indirect costs (value of loss of productivity—described as loss of earnings over presumed number of working years) for both newborn and mother. Intangible ‘benefits’ such as value of newborn death and psychosocial burden caused by false-positive or false-negative results were excluded.

**Analysis:** Assumptions were based on a Finnish prospective study, which showed that prevalence of toxoplasmosis seropositivity among pregnant mothers was 20.3%, incidence of 2.4/1000 seronegative pregnancies, fetomaternal transmission risk of 40% and treatment effectiveness of 50%. Four per cent discount rate was used. The calculations were carried out using a decision analysis.

**Results:** Total financial value of benefit was divided by total costs of the intervention. Sensitivity analysis was conducted. Serological screening for congenital toxoplasmosis (US$30/pregnancy/year) was found to be cheaper than no screening (US$128/pregnancy/year). Net present value (NPV) of savings in Finland was reported at US$2.1 million annually.

**Social return on investment analysis (SROI)** (Alberta Government^23^)

**Background:** Study assessed the SROI of a programme that empowered pregnant homeless women by building their knowledge and skills, providing resources and support to live safer and healthier lives before, during and after pregnancy in Alberta, Canada.

**Costs data:** Based on programme accounting data.

**Benefits data:** Programme data and participant perceptions from interviews, secondary data from other sources such as Ontario 2010 data and Stats Canada report estimate (2002), adjusted to 2010. Benefits looked at 19 months of outcomes data. Based on insights from key stakeholders, project outcomes accounted for were improved maternal health outcomes, improved infant health outcomes, maintained custody of their infants, increased levels of empowerment, improved safety in their environment and personal relationships, positive influence in adopting safer sexual practices and improved housing situation of some clients. The values of all outcomes were presented as financial proxies from secondary data. Benefits were discounted for changes that would have happened without the intervention (dead weight), displacement (eg, how much of another positive activity did program displace)and attribution (what per cent of change was influenced by other factors). Evaluation data and findings from the literature helped determine these discounts. Drop-off rates used were based on estimates of project staff based on their experience. Total financial value of benefit was divided by total costs of the intervention. No sensitivity analysis was conducted.

**Analysis:** Total cost of the program was estimated at US$475 000 and NPV of financial value of benefits created by the intervention is estimated at US$3 438 469. The overall social return of the program was calculated to be US$8.24 for every US$1 invested.

Examples of effectiveness measures that have been used in evaluating MNH interventions include but are not limited to: number of obstetric complications successfully managed, maternal lives saved, stillbirths prevented and newborn lives saved^24^ (box 2). These outcomes are generally expressed in ‘natural units’ that can be measured in principle, as is done in CEA. CEA has been relatively easy to apply, especially as effectiveness is accounted for in natural units, as in the hypothetical example (box 1) and the CEA example^25^ (box 2). This ease of application probably explains why it has been most frequently used in MNH. Researchers only need to report the exact count of the unit of interest. However, a CEA is unidimensional, as such it would be one of both effectiveness measures (‘number of years of mothers saved’ or ‘number of years of newborns saved’) but not both. This could limit the holistic evaluation of MNH interventions or require multiple assessments. Additionally, there is difficulty in comparing across disease conditions or different population groups. Furthermore, CEA does not allow for capturing patient preferences and determination of whether or not an intervention is cost-effective based on a predetermined willingness-to-pay threshold.^27^

However, derived effectiveness measures such as disability-adjusted life years (DALYs) or quality-adjusted life years (QALYs), as is done with CUA, addresses some of these drawbacks. These measures combine morbidity and mortality in one metric^9 26^ (box 2). They take into account the value of the extra length of years gained and the quality of that extra life gained. QALYs are sometimes derived from the valuation of the populations, such as women who benefit from the intervention. For DALYs, health status is not based on self-report (as with QALYs), but on valuations provided by health experts.^28^ However, these experts have focused only on the five leading causes of maternal mortality and their associated morbidities (haemorrhage, sepsis, eclampsia, obstructed labour and abortion). Other direct causes of maternal death such as ectopic pregnancy or indirect causes such as malaria and cardiac disease are not considered.^28^ Thus, DALYs are disease focused, while QALYs are health focused. QALYs are derived from scales tracking physical, mental and social dimensions of health.^29^ However, health encompasses more than these dimensions, particularly when viewed from an extra-welfarist perspective which takes account of broader outcomes.^30^ Indeed MNH interventions usually have broader socioeconomic benefits, such as improved social capital or reduced stigma that need to be captured.^31^ Both QALYs and DALYs have limited applicability in estimating effectiveness and by extension VfM of life-saving interventions such as caesarean section, as both are based on health and disease, respectively.

In CBA and SROI, benefits which refer to ‘effectiveness’ are monetised. Preferences of beneficiaries are taken into account in estimating the valuation of benefits for both, with SROI having the capacity to capture the opinions of a broad range of MNH stakeholders.^32 33^ CBA excludes ‘soft’ or ‘intangible’ outcomes such as psychosocial burden and stigma.^34^ Excluding such outcomes from the ‘cost–benefit analysis’ limits the complete account of the programme benefits, even though they are benefits. SROI, on the other hand, accounts for these other benefits, which are key components of MNH interventions, by using financial proxies. However, collecting these proxies may be challenging.^33^ Overall, both CBA and SROI are particularly sensitive to the assumptions made in their conduct, leaving a risk that both could be manipulated to generate desirable results. This highlights the importance of including sensitivity analyses and clearly documenting any assumptions that are made.^33 35^

To clearly demonstrate effectiveness and cost-effectiveness, assessors need to demonstrate what would have occurred without the intervention. The process is known as establishing the counterfactual and can be done with before-and-after (quasi-experimental) studies, case–control studies, step-wedge design studies or randomised controlled trials. The latter two methods are considered the most robust for evaluation of attribution. When primary effectiveness studies cannot be conducted, some MNH experts have used the Lives Saved Tool (LiST) to assess number of lives saved.^36^ LiST is a module within the demographic software package ‘Spectrum’ which captures national-level data on health status, mortality rates, coverage and effectiveness of over 60 MNH interventions.^37^ The database, ‘Global Value Exchange’ has been developed to standardise sensible financial proxies to account for outcomes for SROI (although this is still a work in progress).^38^

### Choosing a VfM approach for MNH interventions

There are several considerations required in choosing the methods for assessing VfM of an MNH intervention. First, clarity as to the purpose of the VfM assessment and an understanding of the capabilities of the different methods are needed.

Data availability is also a critical consideration. All four approaches collect costs data from programme data or existing secondary data. Most challenges arise with the generation of effectiveness data to populate the cost-effectiveness component. Some MNH studies have used primary data^26^ while others have used available secondary data.^22 23^ Whichever option is used, transparency on how and where the data used was sourced is important.

Furthermore, consideration needs to be given to the capacity of the stakeholders that use VfM information to understand the findings of such analysis. Natural units of CEA are relatively easier to understand as compared with DALYs and QALYs of CUA, though CUA would be more relevant if ‘health’ or ‘disease’ states are the focus of interest. Similarly, monetised outcomes of CBA and SROI are easier to conceptualise for the lay public. However, both CBA and SROI have been described as time consuming and reliant on monetised outcome data which is seldom available.^33 35^

Another key consideration is how the perspectives of beneficiaries are captured. Clearly, perspectives of women are essential to include for evidence-based decision-making to choose MNH interventions that can have a high potential for success. According to the recent synthesis report of the United Nations General Assembly, the focus of the post-2015 agenda rests firmly on leveraging new evaluation measures of subjective well-being that can capture social progress, human well-being, security, justice, equality and sustainability, from the ‘real beneficiaries’.^39^ SROI, though challenging, may prove an invaluable tool to meet this need, given its required engagement of stakeholders throughout the process, including their input as to what costs and benefits should be included for analysis, how long the benefits last for and what portion of the outcomes are attributable to the intervention. If, however, other methods are being used, then deliberate efforts to capture such perspectives are needed.

### Making the VfM judgement

To conduct a comprehensive VfM assessment incorporating all components, one approach is to combine either a CEA or a CUA with a detailed cost analysis and an effectiveness study. The other approach is to use either a CBA or SROI independently. These two approaches would help to generate the quantitative basis for VfM assessments in MNH.

However, the emphasis of a VfM assessment is and should always be on achieving the optimal balance of the 3Es and CE.^21^ As such, after the estimations for each of these elements have been made, it is critical to make a judgement on the overall VfM of the intervention. This ‘judgement’ requires an accompanying narrative providing contextual information explaining any additional considerations as well as justification for choices made. This narrative should incorporate equity considerations around the effectiveness of the intervention,^8 19^ especially as widespread inequalities in MNH programme implementation have been reported.^40^ Such consideration is in line with the new sustainable development goals.^39^ While some have argued that equity can be incorporated into VfM assessments as a dimension of the VfM component—effectiveness—others argue that it should be assessed as a stand-alone fourth ‘E’.^41^ In the MNH area, disaggregated coverage indicators from representative surveys, comparing various segments of the benefitting population, have been used to assess equity (box 1).^40^

Cross-cutting themes such as sustainability, scalability and cultural acceptability should also be assessed and incorporated within the narrative (box 1). These are critical themes to consider, particularly for interventions that aim to improve maternal and newborn health which are often multicomponent, complex interventions involving a range of standards and actual practice.^42 43^ Qualitative engagements with key stakeholders would help provide these critical contextual information and insight for interpreting VfM assessments.^5 44^ For sustainability in particular, establishing the duration for which the impact of the intervention lasts either from evidence in the literature or expert opinion is essential. The duration can also be incorporated in the assessment of the cost-effectiveness of VfM component.

## Conclusion

VfM assessment is very much an evolving science. While the challenges limiting its development are not unique to the area of MNH, they are accentuated within the MNH area, due to the complexity of MNH interventions. To ensure the robustness of VfM assessments in the MNH area, synergy of researchers and practitioners is critical. These assessments would be greatly improved through systematic sharing of experiences and best practices among organisations. Capacity to demonstrate VfM will ensure that MNH interventions can remain competitive for the limited resources in the post-2015 era when more questions regarding VfM will likely continue to be asked.
